# Brain connectome from neuronal morphology

**DOI:** 10.1162/netn_a_00458

**Published:** 2025-07-29

**Authors:** Suhui Jin, Junle Li, Jinhui Wang

**Affiliations:** Institute for Brain Research and Rehabilitation, South China Normal University, Guangzhou, China; Key Laboratory of Brain, Cognition and Education Sciences (South China Normal University), Ministry of Education; Center for Studies of Psychological Application, South China Normal University, Guangzhou, China; Guangdong Key Laboratory of Mental Health and Cognitive Science, South China Normal University, Guangzhou, China; Philosophy and Social Science Laboratory of Reading and Development in Children and Adolescents (South China Normal University), Ministry of Education

**Keywords:** Neural morphology, Brain network, Graph theory, Hub, Comparative connectomics

## Abstract

Single-subject morphological brain networks derived from cross-feature correlation of macroscopic MRI-derived morphological measures provide an important means for studying the brain connectome. However, the validity of this approach remains to be confirmed at the microscopic level. Here, we constructed morphological brain networks at the single-cell level by extending features from macroscopic morphological measures to microscopic descriptions of neuronal morphology. We demonstrated the feasibility and generalizability of the method using neurons in the somatosensory cortex of a rat, neurons over the whole brain of a mouse, and neurons in the middle temporal gyrus (MTG) of a human. We found that interneuron morphological similarity was higher for intra- than interclass connections, depended on cytoarchitectonic, chemoarchitectonic, and laminar classification of neurons (rat), differed between regions with different evolutionary timelines (mouse), and correlated with neuronal axonal projections (mouse). Furthermore, highly connected hub neurons were disproportionately from superficial layers (rat), inhibitory neurons (rat), and subcortical regions (mouse), and exhibited unique morphology. Finally, we demonstrated a more segregated, less integrated, and economic network architecture with worse resistance to targeted attacks for neurons in human MTG than neurons in a mouse’s primary visual cortex. Overall, our method provides an alternative avenue to study neuronal wiring diagrams in brains.

## Author Summary

The brain is a highly complex network spanning multiple spatial scales, yet the organization of brain networks at the single-cell level remains poorly understood. Here, we constructed microscopic morphological brain networks by assessing interneuron similarity based on neuronal morphology for different species. We found that interneuron morphological similarity was correlated with neuronal axonal projections, dependent on neuronal affiliation with respect to cellular and molecular architecture, laminar positioning, and brain area location, and capable of uncovering cross-species differences. Our method complements existing methodology aimed at mapping wiring diagrams in brains at the microscopic level.

## INTRODUCTION

Morphological brain networks depict patterns of interregion relationships in local cortical morphology. Traditionally, morphological brain networks are derived through population-based morphological covariance methods by estimating interregion covariance across participants in a specific morphological feature (Bassett et al., 2008; He et al., 2007; Sanabria-Diaz et al., 2010). These population-based methods, however, limit the broad applicability of morphological brain networks since only one network can be obtained for a participant group. To respond, various methods are developed in recent years to construct morphological brain networks at the single-subject level on the basis of a single image (Jiang et al., 2017; Kong et al., 2015; Li, Wang, et al., 2021; Sebenius et al., 2023; Seidlitz et al., 2018; Tijms et al., 2012; Wang et al., 2016; Yu et al., 2018).

Among the methods for single-subject morphological brain networks, a correlation-based approach grows in popularity, which estimates interregion relationships by calculating Pearson correlation across multiple morphological features (Li et al., 2017; Seidlitz et al., 2018; Zhao et al., 2021). It is found that this across-feature correlation method is robust to methodological variations (e.g., the number of morphological features), reproducible across participants and datasets, and test–retest reliable between scan–rescans (Li et al., 2017; Seidlitz et al., 2018; Zhao et al., 2021). Moreover, the across-feature correlation, method-derived morphological brain networks capture known cortical cytoarchitecture and related gene expression and account for interindividual variation in intelligence (Seidlitz et al., 2018; Zhao et al., 2021), indicative of their biological plausibility. Finally, several studies demonstrate the capacity of the across-feature correlation method to detect network dysfunction in brain disorders, such as major depressive disorder (Li, Seidlitz, et al., 2021) and schizophrenia (Morgan et al., 2019). All these findings indicate that the across-feature correlation method provides a reliable, biologically plausible, and clinically relevant way to study brain networks. However, features used in these studies are all from structural MRI, which measure macroscopic characteristics of brain structure. Whether the across-feature correlation method can be used to construct morphological brain networks at the microscopic level remains largely unknown.

The brain is a complex interconnected network at multiple spatial scales (Sporns et al., 2005). Mapping and characterization of this network at different spatial scales are thus central for a broader understating of brain organization. However, current brain network studies are mainly conducted at the macroscopic level with relatively little knowledge known about the organizational principles of brain networks at the microscopic level. The existing methods to depict microscopic brain networks are based on either electrical or chemical synapses, both of which challenging due to random spontaneous neuronal activity or probabilistic neurotransmitter release (Allen & Stevens, 1994; Faisal et al., 2008; White et al., 2000). Examining the validity of the across-feature correlation method to construct morphological brain networks at the microscopic level may provide an alternative avenue to study neuronal wiring diagrams in brains. Particularly, the across-feature correlation method may provide a unified framework for the multiscale modeling of brain networks.

In this study, we extended the across-feature correlation method to construct morphological brain networks from macroscopic structural MRI-derived measures to microscopic descriptions of neuronal morphology. We first demonstrated the feasibility and generalizability of the method by examining the topological organization of morphological brain networks comprising neurons in the somatosensory cortex of a juvenile rat, neurons over the whole brain of a mouse, and neurons in the middle temporal gyrus (MTG) of an adult human. Then, we evaluated the effects of different cell types, cortical layers, and brain regions on the topological organization of the microscopic morphological brain networks. To further validate the method, we analyzed the relationship between neuronal morphological similarity and axonal projection. Finally, we examined whether the method could capture evolutionary discrepancies by comparing neurons in the MTG of adult human with neurons in the primary visual cortex (VISp) of a mouse. This comparison was informed by findings that human MTG and mouse VISp exhibit similar patterns in gene expression (Berg et al., 2021; Kim et al., 2023) and that histological differences between a human MTG and a mouse temporal association area (a region often used as a comparator for human MTG) are consistent with those observed between human MTG and mouse VISp (Berg et al., 2021).

## MATERIALS AND METHODS

### Neurons

This study included neurons from three datasets. The first dataset (Dataset 1) included neurons from the somatosensory cortex of a juvenile rat, the second dataset (Dataset 2) included whole-brain neurons of a mouse, and the third dataset (Dataset 3) included neurons from the MTG of a human and neurons from the VISp of a mouse. The three datasets were used to demonstrate the feasibility and generalizability of our method. In addition, the Dataset 2 was used to validate our method by comparison with axonal projection data, and the Dataset 3 was used to test whether our method could capture evolutionary discrepancies.

#### Dataset 1.

The rat somatosensory neuron data were from an online public resource of the Blue Brain Project (Markram et al., 2015; https://bbp.epfl.ch/nmc-portal/welcome.html). This dataset contains 1,035 neurons after excluding cloned neurons. These neurons are distributed across five cortical layers and 15 cell types (1 excitatory and 14 inhibitory cell types). Supporting Information Figure S1 shows the number of neurons in each cortical layer (Supporting Information Figure S1A) and cell type (Supporting Information Figure S1B).

#### Dataset 2.

The mouse whole-brain neuron data were available at the SEU-ALLEN Joint Center, Institute for Brain and Intelligence (https://braintell.org/projects/fullmorpho/). This dataset contains 1,741 neurons from 34 brains across 88 brain regions. Supporting Information Figure S1C shows the number of neurons in each region.

#### Dataset 3.

The human MTG neuron data and the mouse VISp neuron data were both from the Allen Institute for Brain Science dataset, which included 101 and 572 neurons after excluding neurons with reconstruction errors, respectively, in cortical layer 2/3 (Berg et al., 2021).

### Morphological Features

For the rat somatosensory neurons, we utilized the L-measure software (Scorcioni et al., 2008) to initially convert Neurolucida ASC format files to the SWC format (Cannon et al., 1998), a standard that has been widely used in various morphological projects, including NeuroMorpho.Org (Ascoli et al., 2007) and BigNeuron (Peng et al., 2015). Then, we used the L-measure software to calculate a total of 42 morphological features for each neuron in each dataset, such as the surface area of soma, the number of stems, and the characteristics of compartments and branches. Detailed explanations and descriptive statistics of the features are provided in Supporting Information Table S1.

### Construction of Microscopic Morphological Brain Networks

To estimate interneuron morphological similarity, we first normalized each morphological feature (*Z*-transformed) to ensure comparability across different morphological features. The normalized morphological features were then correlated (Pearson correlation) between each pair of neurons to generate a 1,035 × 1,035 correlation matrix for the rat somatosensory neurons in the Dataset 1 (Supporting Information Figure S2A, top), a 1,741 × 1,741 correlation matrix for the mouse whole-brain neurons in the Dataset 2 (Supporting Information Figure S2B, top), a 101 × 101 correlation matrix for the human MTG neurons in the Dataset 3 (Supporting Information Figure S2C, top), and a 572 × 572 correlation matrix for the mouse VISp and TEa neurons in the Dataset 3 (Supporting Information Figure S2D, top). Each of the correlation matrices was further thresholded to form a binary network or graph by excluding weak correlations (i.e., set to zero). The binary network model was chosen based on several considerations. First, many widely used graph-based network measures are originally defined for binary networks. While these measures are subsequently adjusted to adapt to weighted networks, the adjustments often require additional assumptions, complicating the interpretation of results. Second, while weighted networks preserve rich information about connectivity strength, they also include weak, nonsignificant connections in them originating from noise that may bring bias to results. Third, binary networks are demonstrated to yield higher test–retest reliability than weighted networks for graph-based network measures derived from human brain networks (Wang et al., 2011, 2016; Yin et al., 2023). Finally, weighted networks are computationally expensive, particularly when the network size (i.e., the number of nodes) is large. Specifically, we utilized a sparsity-based thresholding approach to convert the correlation matrices to binary networks so that the resulting binary networks were as sparse as possible for estimating small-world attributes and meanwhile had no isolated nodes or multiple connected components. Sparsity is defined as the ratio of the number of actually existing edges to the maximum possible number of edges in a network. We first determined the lower limit of the sparsity, below which the small-world attributes were not estimable. That is, for a given binary network, the average degree over all nodes should be larger than 2 × log(*N*), where *N* denotes the number of nodes in the network (Watts & Strogatz, 1998). According to this criterion, the lower sparsity limit was 0.007, 0.004, 0.046, and 0.011 for the rat somatosensory, mouse whole-brain, human MTG, and mouse VISp microscopic morphological brain networks, respectively. However, we found that the binary networks derived at these sparsities contained isolated nodes or multiple connected components, which made the calculation of graph-based network measures problematic and unreliable. Thus, we continuously increased the sparsity until all nodes just formed one connected component for each binary network. This procedure finally resulted in a binary network composed of 38,984 edges (sparsity = 0.073) for the rat somatosensory morphological brain network, a binary network composed of 139,705 edges (sparsity = 0.092) for the mouse whole-brain morphological brain network, a binary network composed of 468 edges (sparsity = 0.093) for the human MTG morphological brain network, and a binary network composed of 10,109 edges (sparsity = 0.062) for the mouse VISp morphological brain network. In these binary networks, the minimum correlation coefficients were 0.449, 0.521, 0.497, and 0.413, corresponding to significance levels of 0.003, 0.000, 0.001, and 0.006, respectively. Notably, negative correlations were excluded prior to the thresholding procedure due to their ambiguous biological relevance and detrimental effects on test–retest reliability of grapy-based network measures (Wang et al., 2011).

### Graph-Based Network Analysis of Microscopic Morphological Brain Networks

For the binary microscopic morphological brain networks derived above, several graph-based network measures were calculated to characterize their topological organization at global, nodal, and connectional levels. Detailed descriptions and interpretations of the network measures can be found elsewhere (Rubinov & Sporns, 2010; Wang et al., 2011). All network analyses were performed with the GRETNA toolbox (Wang et al., 2015).

#### Small-world attributes.

Small-world attributes, including clustering coefficient and characteristic path length, are originally proposed by Watts and Strogatz (1998). Clustering coefficient quantifies the local interconnectivity of a network, and characteristic path length is an indicator of the overall routing efficiency of a network. These two attributes were normalized by the corresponding mean of random networks generated using a topological rewiring method to ensure the same degree distributions as the real networks (Maslov & Sneppen, 2002). Typically, a network is said to be small-world if it has higher clustering coefficient than but approximately equal characteristic path length to matched random networks (i.e., normalized clustering coefficient, γ > 1 and normalized characteristic path length, λ ~ 1; Watts & Strogatz, 1998). These two conditions can be further summarized by a scalar called small-worldness, σ = γ/λ > 1 for a small-world network. In this study, 100 random networks were generated, consistent with numerous previous studies (e.g., Sun et al., 2024; van den Heuvel & Sporns, 2011). However, given that the network size (i.e., the number of nodes) varied largely among the microscopic morphological brain networks included in this study, we examined whether 100 random networks were enough for reliably estimating the small-world organization. In theory, more random networks are required for larger networks. Thus, we recalculated normalized clustering coefficient and normalized characteristic path length for the mouse whole-brain microscopic morphological network with the number of random networks varying from 100 to 1,000 (interval = 100). This network was chosen because it contained the most nodes (i.e., the largest network size) among all microscopic morphological brain networks included in this study. We found small fluctuations in the estimates of normalized clustering coefficient and normalized characteristic path length (Supporting Information Figure S3), indicating that 100 random networks are appropriate for reliably estimating the small-world organization of microscopic morphological brain networks in this study.

#### Cost efficiency.

For a binary network, cost is defined as the total number of edges divided by the maximum possible number of edges in the network, which measures how expensive it is to build the network (Latora & Marchiori, 2001). Global efficiency is the inverse of the harmonic mean of the minimum path length between all pairs of nodes in a network, which reflects the overall ability of parallel information transmission (Latora & Marchiori, 2001). Cost efficiency is the difference between global efficiency and cost, which is positive for an economical network (Achard & Bullmore, 2007).

#### Nodal degree, degree distribution, and hubs.

To evaluate the role of individual neurons in the microscopic morphological brain networks, we computed nodal degree, which is defined as the total number of edges linked to a node. Previous studies have shown that small-world brain networks can follow different forms of degree distribution (Achard et al., 2006; Eguíluz et al., 2005; Hagmann et al., 2008; He et al., 2007; Wang et al., 2009), which are related to different network behaviors, such as resilience to random errors or hub attacks (Achard et al., 2006; Albert et al., 2000; Amaral et al., 2000). Thus, different models (i.e., power law, exponential, and exponentially truncated power law) were used to fit the degree distributions of the microscopic morphological brain networks. Consistent with previous brain network studies (e.g., Cheng et al., 2015; Ruan et al., 2023), nodes in the top 10% of the ranked nodal degree were identified as hub nodes.

#### Edge betweenness and bridges.

Edge betweenness was calculated to explore the role of each edge in the microscopic morphological brain networks, which is defined as the fraction of shortest paths in a network that pass through a given edge. Edge betweenness captures the influence of an edge on information flow between nodes in a network. Similar to the definition of hub nodes, edges in the top 10% of the ranked edge betweenness were identified as bridge edges.

#### Network resilience.

To assess the resilience of the microscopic morphological brain networks to lesions on nodes and edges, we performed the following simulation analyses, as in our previous study (He et al., 2009). Briefly, nodes or edges were removed one by one in a manner of random failure and targeted attack, respectively, followed by the recalculation of cost efficiency of the networks consisting of the rest of nodes and edges. For the random failure, a node or edge was randomly selected and removed, while for the targeted attack, the node or edge with the highest degree or betweenness was removed.

### Effects of Different Classes on Microscopic Morphological Brain Networks

We performed a series of analyses to explore the effects of different classes (i.e., cortical layers or cell types for the Dataset 1 and brain regions for the Dataset 2) on the microscopic morphological brain networks. Notably, the neurons were not uniformly distributed across the cell types and brain regions, with some cell types and brain regions containing very few neurons. These cell types and brain regions were thus excluded in the analyses. Specifically, a cutoff of 65 neurons was used to filter out cell types. This cutoff was chosen to ensure the inclusion of excitatory neurons while minimizing the imbalance between the excitatory and inhibitory neurons as much as possible. For brain regions, a smaller cutoff of 30 neurons was used given that the number of brain regions was far more than the number of cell types. This cutoff was determined because sample size larger than 30 often satisfies the conditions of the central limit theorem, allowing for stable and robust statistical inferences (Roscoe, 1975). Consequently, the analyses were performed for 8 out of the 15 cell types for the Dataset 1 (i.e., nest basket cell [NBC], double Bouquet cell [DBC], bipolar cell [BP], large basket cell [LBC], Martinotti cell [MC], neurogliaform cell [NGC], bitufted cell [BTC], and pyramidal cell [PC]) and 13 out of the 88 brain regions for the Dataset 2 (i.e., ventral posteromedial nucleus [VPM], caudoputamen [CP], primary somatosensory area, mouth [SSp-m], ventral posterolateral nucleus [VPL], lateral geniculate complex, dorsal part [LGd], supplemental somatosensory area [SSs], primary motor area [MOp], primary somatosensory barrel cortex [SSp-bfd], medial geniculate complex [MG], secondary motor area [MOs], reticular nucleus [RT], primary somatosensory area, upper limb [SSp-ul], and VISp).

First, we calculated the small-world attributes and cost efficiency for the binary subnetworks composed of neurons within each class to test whether the subnetworks also exhibited small-world organization and economy.

Second, we examined whether the identified hub nodes were disproportionately located in specific classes. Specifically, we defined a disproportionate contribution coefficient, *DCC*, for each class as:

DCCi=NhubsiNhubsNnodesiNnodes,

where *N*_*nodes*_ is the total number of nodes, *N*_*hubs*_ is the total number of hub nodes, $N_{\text{nodes}}^{\left(i\right)}$ is the number of nodes in a given class, and $N_{\text{hubs}}^{\left(i\right)}$ is the number of hub nodes in the given class. A *DCC* larger than 1 indicates a disproportionate distribution of hub nodes in a class. Similarly, the *DCC* was calculated to examine whether the bridge edges were disproportionately related to connections within the same class or between different classes and connections within or between specific classes.

Finally, we examined the differences in the interneuron morphological similarity between intra- and interclass connections in the binary microscopic morphological brain networks. Given that the number of interclass connections was much greater than the number of intraclass connections (cortical layer: 27,084 vs. 11,900; cell type: 23,744 vs. 15,240; brain region: 118,502 vs. 21,203), we utilized a bootstrapping-like method, which was explained with the cortical layer as an example. First, we calculated the mean morphological similarity for connections within the same cortical layers, *S*_*intra*_. Then, we randomly selected the same number of connections (i.e., 11,900) and calculated their mean morphological similarity, *S*_*net*_. After 10,000 repetitions of the random procedure, a Z score was finally calculated as:

$$Z=\frac{S_{\text{intra}}-\text{mean}\left(S_{net}\right)}{\text{standard deviation}\;\left(S_{net}\right)},$$

with the corresponding *p* value determined by a standard normal distribution.

Following the comparisons between the intra- and interclass connections, we examined whether different classes exhibited different levels of intraclass morphological similarity using a nonparametric permutation testing approach based on an *f*-statistic derived from an analysis of variance. If a significant group effect was observed, post hoc pairwise comparisons were further performed (*t*-statistic-based permutation testing). The nonparametric permutation testing approach was used because it does not assume normality of the data and is particularly well suited for unbalanced designs where group sizes differ significantly. Multiple comparisons were corrected using a false discovery rate (FDR) procedure at the level of *q* < 0.05.

### Relationship Between Morphological Features and Hub Nodes in Microscopic Morphological Brain Networks

To explore whether hub nodes are associated with unique morphological features, we compared each morphological feature between hub and nonhub nodes in the microscopic brain networks using a bootstrapping-like approach as mentioned above. First, for a given morphological feature, we calculated its mean for the hub nodes, *M*_*hub*_. Then, we randomly selected the same number of neurons as the hub nodes and calculated their mean for the given morphological feature, *M*_*net*_. After 10,000 repetitions of these random procedures, a Z score was calculated as:

$$Z=\frac{M_{hub}-\text{mean}\left(M_{net}\right)}{\text{standard deviation}\;\left(M_{net}\right)}.$$

Finally, multiple comparisons were corrected across the 42 morphological features using the FDR procedure at the level of *q* < 0.05.

### Relationship Between Neuronal Morphological Similarity and Axonal Projections

In this study, we estimated interneuron connectivity in terms of morphological similarity. An important question was whether the morphological similarity reflected neuronal axonal projections. To answer this question, we compared the mouse whole-brain microscopic morphological brain network with publicly available axonal projection data of neurons in the Dataset 2 (Peng et al., 2021). The Dataset 2 contained 1,741 neurons from 34 brains across 88 brain regions. For 1,740 out of the 1,741 neurons, Peng et al. (2021) released their axonal projection profiles to 315 brain regions in their ipsilateral brain hemisphere and the same 315 brain regions in their contralateral brain hemisphere. The 1,740 neurons were distributed in 87 regions, which were a subset of the 315 regions. Thus, we constructed the axonal projection matrix among the 87 regions. Specifically, for a given region, its axonal projection profile to the 87 regions in its ipsilateral brain hemisphere was calculated by averaging the axonal projection profiles of all neurons within the given region to the 87 regions in its ipsilateral brain hemisphere. This resulted in an 87 × 87 matrix, with an element *P*_*ij*_ denoting the axonal projection from region *i* to region *j* that was located in the same brain hemisphere with region *i*. Analogously, an 87 × 87 matrix was obtained to represent the axonal projections from each of the 87 regions to the 87 regions in their contralateral brain hemisphere. These two 87 × 87 matrices were further averaged to derive a more representative axonal projection matrix among the 87 regions. Finally, the interregion axonal projection matrix was symmetrized by addition with its transposed matrix. Notably, since there were many zeros in the neuron-to-region axonal projection data, only nonzero elements were used to calculate the mean interregion axonal projections. Meanwhile, an 87 × 87 interregion morphological similarity matrix was obtained to represent the mean morphological similarity between each pair of the 87 regions. Again, only nonzero elements in the binary mouse whole-brain microscopic morphological brain network were used to estimate the mean interregion morphological similarity.

We used four methods to analyze the relationship between the interregion morphological similarity matrix and the interregion axonal projection matrix. First, we counted the number of nonzero (i.e., the presence of a morphological connection) and zero (i.e., the absence of a morphological connection) values in the interregion morphological similarity matrix separately for brain pairs with and without axonal projections, and performed a chi-square test. Then, we compared the morphological similarity between brain regions interconnected by axonal projections with that between brain regions not interconnected by axonal projections with a nonparametric permutation testing approach based on a *t*-statistic derived from a two-sample *t* test. Subsequently, we calculated the cosine similarity for each pair of regions separately in terms of their morphological similarity profiles and axonal projection profiles, and examined the spatial correspondence of the two resulting matrices (Pearson correlation across edges). Instead of the original values of morphological similarity and axonal projection, the use of cosine similarity ensured that the variables used for the correlation calculation had the same scale and nature. Finally, we compared hubs between the interregion morphological similarity matrix and the interregion axonal projection matrix. First, each matrix was thresholded using the same procedure as for the microscopic morphological brain networks. This procedure resulted in a sparsity of 0.381 (1,426 edges) for the interregion morphological similarity matrix and a sparsity of 0.214 (800 edges) for the interregion axonal projection matrix. To improve the comparability, both the interregion morphological similarity and axonal projection matrices were thresholded at sparsity = 0.381 (1,426 edges). For the two resulting binary networks, we calculated the nodal degree for each region and define hubs again as nodes in the top 10% of the ranked nodal degree.

### Cross-Species Comparison of Microscopic Morphological Brain Networks

To explore whether our method can capture evolutionary discrepancies, we compared the small-world attributes (γ, λ, and σ), cost efficiency, and resilience to targeted attacks on nodes and edges (indexed by the area under the cost efficiency curve with continuous removal of nodes and edges) between the mouse VISp and human MTG microscopic morphological brain networks (Dataset 3) via a nonparametric permutation testing approach. For a given network measure, we first calculated its difference between the mouse VISp and human MTG morphological brain networks, *D*_*real*_. Then, all neurons in the mouse VISp and human MTG were mixed together and randomly reallocated into two groups (572 vs. 101 neurons). This randomization procedure was repeated 10,000 times to create 10,000 pairs of randomized groups. For neurons in each pair of randomized groups, morphological brain networks were constructed and thresholded in the same manner as described above, followed by calculation of the network measure. The difference in the network measure between the two randomized groups was recorded as *D*_*rand*_. Finally, for the given network measure, its actually observed difference was benchmarked against the null distribution of its difference derived from the 10,000 pairs of randomized groups as:

$$Z=\frac{D_{\text{real}}-\text{mean}\left(D_{\mathrm{rand}}\right)}{\text{standard deviation}\;\left(D_{\mathrm{rand}}\right)}.$$

## RESULTS

### Stability and Robustness of the Microscopic Morphological Brain Networks

In this study, we constructed the microscopic morphological brain networks by calculating the Pearson correlation across 42 morphological features between neurons. We first assessed the stability and robustness of the microscopic morphological brain networks. Using a leave-one-feature-out approach, we found that the microscopic morphological brain networks were stable as demonstrated by the high Pearson correlation coefficients between the interneuron correlation matrices derived from all morphological features and those generated with one feature removed (Supporting Information Figure S4A). To evaluate whether the microscopic morphological brain networks were robust to variations in the numbers of features, we randomly selected different numbers of morphological features (from 21 to 39 with an interval of 3) to re-estimate interneuron correlation matrices (10,000 times). We found that the correlation matrices became increasingly similar to the interneuron correlation matrices derived from all morphological features as the number of morphological features increased (Supporting Information Figure S4B). Particularly, when more than 30 morphological features were used, the resulting interneuron correlation matrices exhibited high Pearson correlation coefficients (approximate or greater than 0.8) with those derived from all morphological features. These findings suggest that microscopic morphological brain networks can be constructed more precisely using more features per neuron.

### Small-Worldness of the Microscopic Morphological Brain Networks

**Figure 1.**
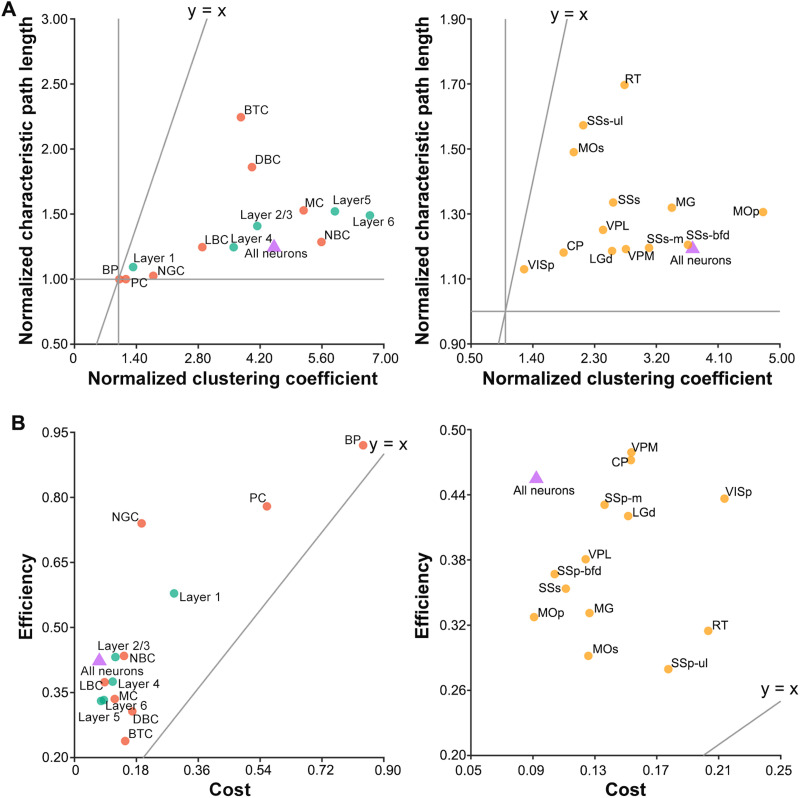
Small-worldness and cost efficiency of the microscopic morphological brain networks. (A) Compared with random networks, the morphological brain networks exhibited higher clustering coefficient but approximately equal characteristic path length, suggesting a small-world organization (purple triangle, the left for the rat somatosensory and the right for the mouse). (B) The microscopic morphological brain networks had a higher efficiency than cost, indicating economical organization (purple triangle, right, the left for the rat somatosensory and the right for the mouse). These organizational characteristics were also observed for neurons within each cortical layer (turquoise circles), neurons of each cell type (orange circles), and neurons of each brain region (yellow circles).

Compared with matched random networks, the rat somatosensory, mouse whole-brain, human MTG, and mouse VISp microscopic morphological brain networks showed higher clustering coefficients (γ = 4.513, 3.728, 2.931, and 3.412, respectively) and approximately equal characteristic path lengths (λ = 1.245, 1.193, 1.339, and 1.269, respectively), resulting in the small-worldness σ = 3.363, 3.126, 3.537, and 2.690, respectively. That is, the microscopic morphological brain networks exhibit typical small-world organization regardless of the species. Further analyses revealed that neurons within each cortical layer and neurons of each cell type in the rat somatosensory cortex (Figure 1A, left), and neurons within each brain region in the mouse (Figure 1B, left) also showed the small-world organization.

### Cost Efficiency of the Microscopic Morphological Brain Networks

The rat somatosensory, mouse whole-brain, human MTG, and mouse VISp microscopic morphological brain networks exhibited positive cost efficiency of 0.350, 0.363, 0.246, and 0.325, respectively, indicating the economy. The economy was also observed for neurons within each cortical layer and neurons of each cell type in the rat somatosensory cortex (Figure 1A, right), and neurons within each brain region in the mouse (Figure 1B, right).

### Hub Nodes in the Microscopic Morphological Brain Networks

The degree distributions of the rat somatosensory, mouse whole-brain, and human MTG microscopic morphological brain networks were best fitted by an exponentially truncated power law, P(k) ~ *k*^*α*−1^*e*^−*k*/*k*_*c*_^, with estimated exponents of *α* = 1.316, 1.301, and 1.519, respectively, and cutoff degrees of *k*_*c*_ = 70.021, 126.021, and 5.160, respectively (Supporting Information Figure S5). That is, there is an exponential decline in the probability of a node with a degree greater than ~70, 126, and 5 in the rat somatosensory, mouse whole-brain, and human MTG microscopic morphological brain networks, respectively. This type of degree distribution allows the existence of some central neurons with many connections but prevents the appearance of huge hubs with very many connections in the microscopic morphological brain networks.

**Figure 2.**
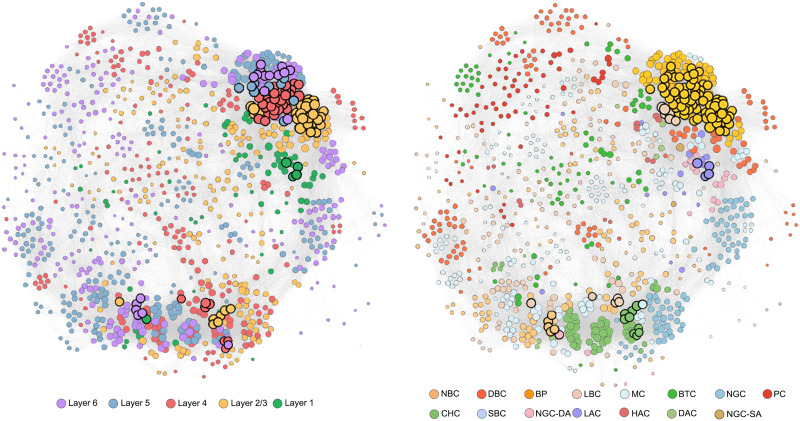
Topological representation of the rat somatosensory microscopic morphological brain network. Neurons are represented as nodes, and interneuron morphological similarities are represented as edges. Nodal sizes correspond to the nodal degree. The nodes that rank within the top 10% in terms of the nodal degree are classified as hub nodes and are visually distinguished with bold black circles. All remaining nodes, which are visually represented by nonbold circles, are classified as nonhub nodes. The left displays the topological representation of the network with respect to the cortical layers, while the right shows the network representation according to the cell types. CHC, chandelier cell; SBC, small basket cell; NGC-DA, NGC with dense axonal arborization; LAC, large axon cell; DAC, descending axon cell; NGC-SA, neurogliafomm cell with slender axonal arborization.

**Figure 3.**
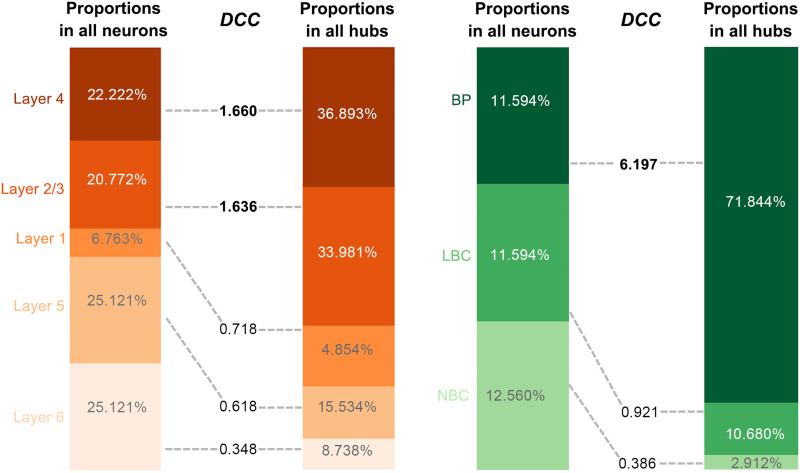
Distribution of hub nodes in the rat somatosensory microscopic morphological brain network. In terms of cortical layers, hub nodes were mainly located in layer 4 and layer 2/3 (left). In terms of cell types, all hub nodes were inhibitory neurons and were mainly BP neurons (right). *DCC*, disproportionate contribution coefficient.

Figure 2 visualizes the 103 hub nodes by mapping the rat somatosensory microscopic morphological brain network with the Gephi software (Bastian et al., 2009), with its layout determined by a Fruchterman Reingold algorithm (Fruchterman & Reingold, 1991). We found that the hub nodes were mainly located in layer 4 (36.893%) and layer 2/3 (33.981%). These proportions were greater than the ratios of neurons in these layers to all neurons, resulting in *DCC* values of 1.660 and 1.636, respectively (Figure 3, left). For cell types, we found that all hub nodes were inhibitory neurons. Furthermore, the hub nodes were mainly BP neurons (71.844%). This proportion was far greater than the ratio of BP neurons to all neurons, resulting in a *DCC* of 6.197 (Figure 3, right). Given that all cortical layers can be generally categorized into superficial (layer 1 and layer 2/3) and deep (layer 4, layer 5, and layer 6) layers, and all cell types can be generally categorized into excitatory (PC) and inhibitory (NBC, DBC, BP, LBC, MC, NGC, and BTC) neurons, we also examined the *DCC* of the hub nodes to each of these categories. A larger-than-1 *DCC* was found for the superficial (*DCC* = 1.410) but not deep (*DCC* = 0.871) layers and for the inhibitory (*DCC* = 1.184) but not excitatory (*DCC* = 0) neurons.

For the mouse whole-brain microscopic morphological brain network, we found that the hub nodes were disproportionately located in the CP (*DCC* = 1.609), VISp (*DCC* = 1.563), VPM (*DCC* = 1.429), LGd (*DCC* = 1.283), and RT (*DCC* = 1.177; Supporting Information Figure S6, left). When categorizing the brain regions into isocortical (SSp-m, SSs, MOp, SSp-bfd, MOs, SSp-ul, and VISp) and subcortical (VPM, CP, VPL, LGd, MG, and RT) structures, a *DCC* larger than 1 was observed only for the subcortical regions (*DCC* = 1.408; isocortex regions: *DCC* = 0.565).

### Bridge Edges in the Microscopic Morphological Brain Networks

**Figure 4.**
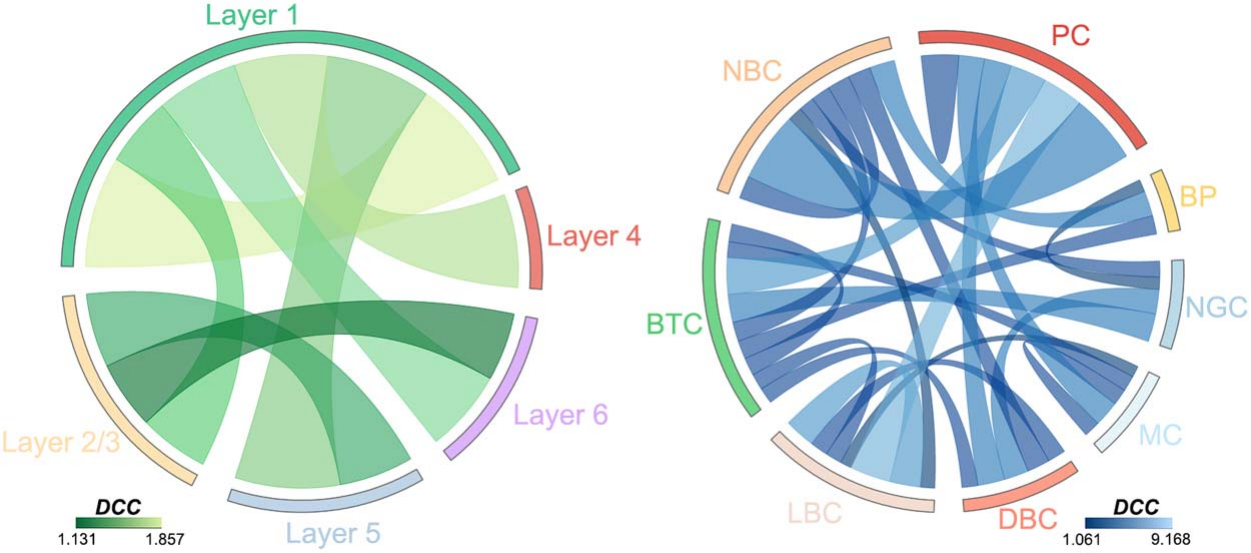
Distribution of bridge edges in the rat somatosensory microscopic morphological brain network. The bridge edges preferred connections linking different classes to connections within the same class. Disproportionate contributions to the bridge edges were made by connections between six pairs of cortical layers (left) and 20 pairs of cell types (right). Notably, among the pairs of classes, the majority were associated with layer 1 (left) or PC and NBC neurons (right).

A total of 3,898 connections were identified as bridge edges in the rat somatosensory microscopic morphological brain network. The bridge edges were more likely to be connections linking different classes (cortical layer: *DCC* = 1.030; cell type: *DCC* = 1.320) than connections within the same classes (cortical layer: *DCC* = 0.931; cell type: *DCC* = 0.501). This preference was further validated by examining the *DCC* for connections within each and between each pair of classes (Figure 4). Specifically, we found that connections between six pairs of cortical layers and 20 pairs of cell types made disproportionate contributions to the bridge edges (i.e., *DCC* > 1), whereas connections within one cortical layer and five cell types had *DCC* > 1. Notably, among the pairs of classes with *DCC* > 1, most were related to layer 1 (4, 66.667%) or PC (5, 25%) and NBC (5, 25%) neurons.

For the mouse whole-brain microscopic morphological brain network, a total of 13,970 connections were identified as bridge edges. Similarly, the bridge edges were more likely to be connections linking different classes (brain regions: *DCC* = 1.026) than connections within the same classes (brain regions: *DCC* = 0.856). This preference was further validated by examining the *DCC* for connections within each and between each pair of classes (Supporting Information Figure S6, right). That is, connections between 46 pairs of brain regions made disproportionate contributions to the bridge edges (i.e., *DCC* > 1), whereas connections within only seven brain regions had *DCC* > 1 (Supporting Information Figure S6, right).

### Resistance of the Microscopic Morphological Brain Networks

As expected, targeted attacks on nodes with high degree caused a more rapid deterioration in the cost efficiency of the rat somatosensory (Supporting Information Figure S7A, top), mouse whole-brain (Supporting Information Figure S7B, top), and human MTG (Supporting Information Figure S7C, top) microscopic morphological brain networks than random failures of nodes. Similarly, targeted attacks on edges with high betweenness resulted in a faster decline in the cost efficiency of the microscopic morphological brain networks than random errors of edges (Supporting Information Figure S7A, bottom; Supporting Information Figure S7B, bottom; Supporting Information Figure S7C, bottom). Interestingly, targeted attacks on edges with high betweenness appeared to lead to larger degenerative effects on the cost efficiency than targeted attacks on nodes with high degree in the rat somatosensory, mouse whole-brain, and human MTG microscopic morphological brain networks when the same fractions of nodes and edges were removed. For example, for the rat somatosensory microscopic brain network, the cost efficiency was reduced from 0.350 to 0.093 when 10% edges were attacked, whereas the cost efficiency remained nearly unchanged when the same proportion of nodes were removed. Further statistical comparisons revealed that when the same fractions of edges and nodes were targeted (1%–100%, 1% intervals), targeting edges led to greater reductions in the cost efficiency of the microscopic morphological brain networks regardless of the species (two-sample *t* test, *p* < 0.001).

### Effects of Cortical Layers, Cell Types, and Brain Regions on the Microscopic Morphological Brain Networks

For the rat somatosensory microscopic morphological brain network, a significantly higher morphological similarity was observed for intra- than interclass connections (cortical layers: *Z* = 4.991, *p* < 0.001; cell types: *Z* = 5.010, *p* < 0.001). For intraclass connections, significantly different levels of morphological similarity were further observed between different classes (cortical layers: *p* < 0.001; cell types: *p* < 0.001). Post hoc comparisons revealed a continuous increase in the intraclass morphological similarity from the superficial to deep layers (*p* < 0.05, FDR corrected; Supporting Information Figure S8A) and the highest intraclass morphological similarity for the NGC neurons and lowest intraclass morphological similarity for the MC neurons (Supporting Information Figure S8C).

For the mouse whole-brain microscopic morphological brain network, a significantly higher morphological similarity was observed for intra- than interclass connections (brain regions: *Z* = 45.912, *p* < 0.001), and the levels of intraclass morphological similarity differed significantly among classes (brain regions: *p* < 0.001), with the highest value within the SSp-ul and the lowest value within the MOs (Supporting Information Figure S8B).

**Figure 5.**
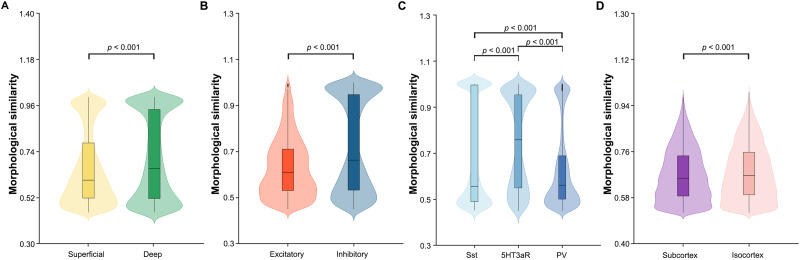
Effects of cortical layers, cell types, and brain regions on morphological similarity in the microscopic morphological brain networks. Significantly higher intraclass morphological similarity was observed for the deep than superficial layers (A), for the inhibitory than excitatory neurons (B), and for the isocortical than subcortical structures (D). Figure 5C shows the differences in morphological similarity between different subtypes of inhibitory neurons. The highest morphological similarity was observed for connections linking neurons within the 5HT3aR, followed by the PV and Sst.

To better understand the trends in the post hoc results, we compared the intraclass morphological similarities between the superficial and deep layers, excitatory and inhibitory neurons, and isocortical and subcortical structures. Significantly higher intraclass morphological similarity was observed for the deep than superficial layers (*Z* = 13.223, *p* < 0.001; Figure 5A), for the inhibitory than excitatory neurons (*Z* = 23.578, *p* < 0.001; Figure 5B), and for the isocortical than subcortical structures (*Z* = 15.770, *p* < 0.001; Figure 5D). Since inhibitory neurons can be classified into different subtypes according to their expressed molecular markers (calcium-binding protein parvalbumin [PV]: LBC and NBC; neuropeptide somatostatin [Sst]: MC; ionotropic serotonin receptor [5HT3aR]: BTC, DBC, and BP; Tremblay et al., 2016), we also compared the intraclass morphological similarity between the subtypes of inhibitory neurons. The highest morphological similarity was observed for connections linking neurons within the 5HT3aR, followed by the PV and Sst (*p* < 0.05, FDR corrected; Figure 5C).

### Unique Neuronal Morphology of Hub Nodes in the Microscopic Morphological Brain Networks

**Figure 6.**
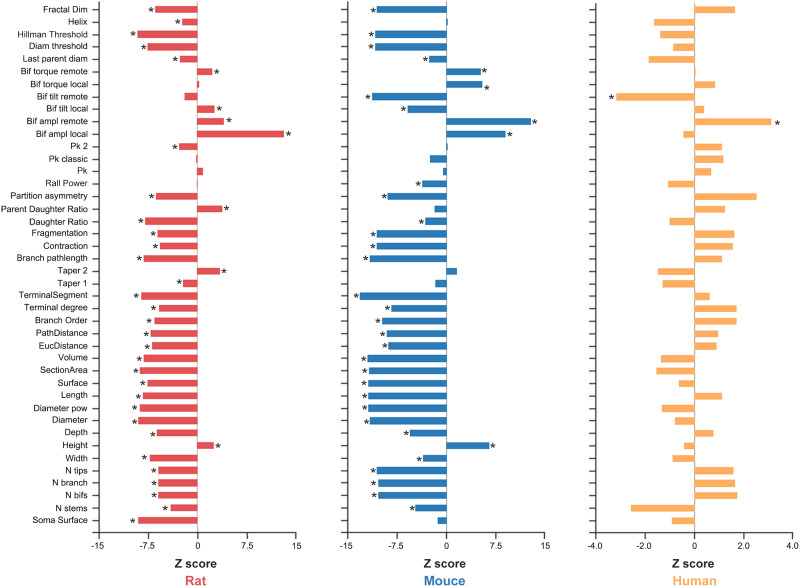
Differences in neuronal morphology between hub and nonhub nodes. For both the rat somatosensory and mouse whole-brain microscopic morphological brain networks, significant differences were observed in most neuronal morphological features between hub and nonhub nodes. For the human MTG microscopic morphological brain network, however, only two neuronal morphological features differed significantly between hub and nonhub nodes. Notably, significantly larger bifurcation amplitude angles between the two bifurcations or terminations at the end of the daughter branches were consistently observed across all microscopic morphological brain networks. See Supporting Information Table S1 for a detailed description of the morphological features. *p < 0.05, FDR corrected.

For the rat somatosensory (Figure 6, left) and mouse whole-brain (Figure 6, middle) microscopic brain networks, the hub nodes exhibited significant differences in most neuronal morphological features from nonhub nodes, such as higher neuronal height and fewer branches (*p* < 0.05, FDR corrected). Moreover, the differences displayed largely similar patterns between the rat and mouse networks. For the human MTG microscopic morphological brain network, however, only two neuronal morphological features showed significant differences between the hub and nonhub nodes (*p* < 0.05, FDR corrected; Figure 6, right). Interestingly, significantly larger bifurcation amplitude angles between the two bifurcations or terminations at the end of the daughter branches were consistently observed for the hub nodes than for the nonhub nodes in the rat somatosensory (*Z* = 4.015, *p* < 0.001), mouse whole-brain (*Z* = 12.749, *p* < 0.001), and human MTG (*Z* = 3.067, *p* = 0.002) microscopic morphological brain networks.

### Associations of Neuronal Morphological Similarity with Axonal Projections

**Figure 7.**
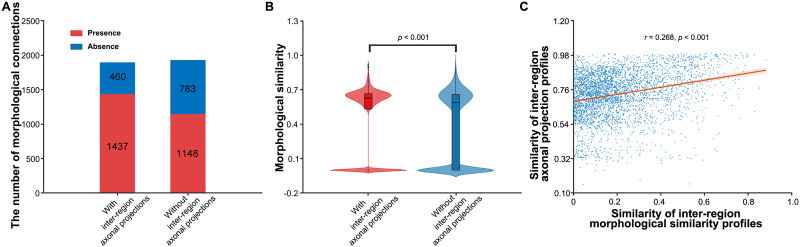
Relationship between neuronal morphological similarity and axonal projections in the mouse whole-brain microscopic morphological brain network. (A) Significantly more morphological connections were observed between brain pairs with axonal projections than between brain pairs without axonal projections. (B) Brain pairs interconnected by axonal projections showed significantly higher morphological similarity than those without such interconnections. (C) There was a significantly positive correlation in the pattern of interregion similarity of connectivity profiles between morphological similarity and axonal projection.

In the mouse whole-brain microscopic morphological brain network, significantly more morphological connections existed between brain pairs with axonal projections than between those without axonal projections (*χ*^2^ = 113.441, *p* < 0.001; Figure 7A). Further comparison of the morphological similarity revealed significantly higher values between brain pairs interconnected by axonal projections than those not interconnected (*p* < 0.001; Figure 7B). Furthermore, a significantly positive correlation was observed in the pattern of interregion similarity of connectivity profiles between morphological similarity and axonal projection (*r* = 0.268, *p* < 0.001; Figure 7C). Finally, while we found a significantly positive correlation for nodal degree between the mouse interregion morphological similarity and axonal projection networks (*r* = 0.317, *p* = 0.003), only one region (i.e., the central amygdalar nucleus) was commonly identified as a hub.

### Differences Between the Mouse VISp and Human MTG Microscopic Morphological Brain Networks

**Figure 8.**
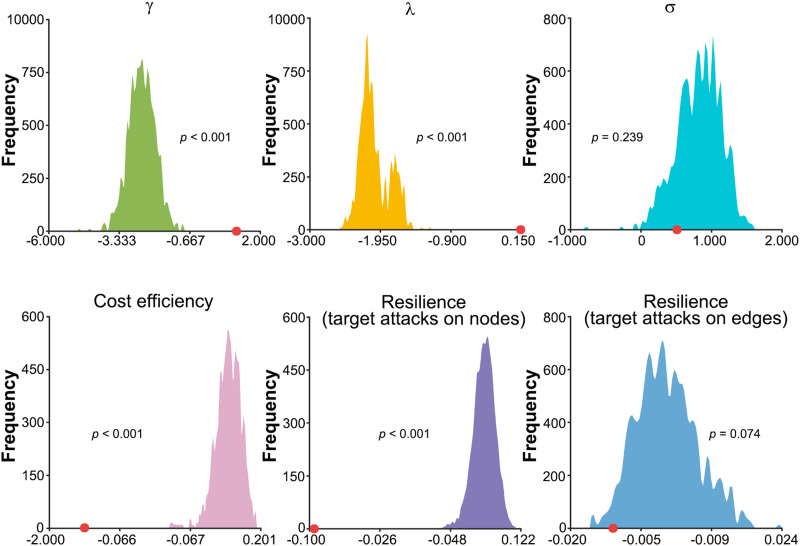
Differences between the mouse VISp and human MTG morphological brain networks. Compared with the mouse VISp morphological brain network, the human MTG morphological brain network exhibited a more segregated (higher γ), less integrated (higher λ), and economic (lower cost efficiency) network architecture with worse resistance to targeted attacks on nodes and edges (lower area under the cost efficiency curve with continuous removal of nodes and edges). The red circles indicate real observations. γ, normalized clustering coefficient; λ, normalized characteristic path length; σ, small-worldness scalar.

Compared with the mouse VISp morphological brain network, the human MTG morphological brain network exhibited significantly higher γ (*Z* = 7.039, *p* < 0.001) and λ (*Z* = 9.960, *p* < 0.001) but lower cost efficiency (*Z* = −11.304, *p* < 0.001) and resistance to targeted attacks on nodes (*Z* = −21.311, *p* < 0.001) and edges (*Z* = −1.786, *p* = 0.074; Figure 8). No significant differences were observed in σ (*p* > 0.05).

### Effects of Feature Redundancy on Microscopic Morphological Brain Networks

In this study, we found removal of some neuronal morphological features does not change interneuron similarity matrices obviously (Supporting Information Figure S4A). This finding indicates the existence of redundant features. Thus, we calculated Pearson correlation between each pair of neuronal morphological features for each species, resulting in four 42 × 42 correlation matrices. Strongly correlations were observed between certain pairs of neuronal morphological features regardless of the species, such as fragmentation and partition asymmetry (Supporting Information Figure S9).

To examine the extent to which our results were affected by the feature redundancy, we applied a principal component analysis (PCA) to reduce the redundancy among the 42 neuronal morphological features (Abdi & Williams, 2010). PCA transforms the data into a set of new orthogonal variables by identifying the directions (principal components) along which the variation in the data is maximal (Ringnér, 2008). We retained the first 20 principal components for each species that collectively explained more than 95% of the variance in the data. Using the 20 principal components, we constructed microscopic morphological brain networks and examined the similarity of the resultant networks with those constructed with all 42 neuronal morphological features by calculating Pearson correlation in interneuron morphological similarity across all edges. Extremely high correlations were observed: *r* = 0.921, 0.935, 0.924, and 0.936 for the rat somatosensory, mouse whole-brain, human MTG, and mouse VISp microscopic morphological networks. These findings indicate that all results from graph-based topological analyses reported above are robust against redundant features as they are derived from binary networks, which are insensitive to global shift in connectivity weights.

We re-examined the effects of cortical layers, cell types, and brain regions on interneuron morphological similarity using the microscopic morphological brain networks constructed with the 20 principal components. The results were largely comparable with those derived from the microscopic morphological brain networks constructed with all 42 neuronal morphological features: (a) A significantly higher morphological similarity was observed for the intra- than interclass connections (cortical layers: *Z* = 32.358, *p* < 0.001; cell types: *Z* = 39.074, *p* < 0.001; brain regions: *Z* = 62.097, *p* < 0.001); (b) a significantly higher intraclass morphological similarity was observed for the deep than superficial layers (*Z* = 14.913, *p* < 0.001), for the inhibitory than excitatory neurons (*Z* = 23.185, *p* < 0.001), and for the isocortical than subcortical structures (*Z* = 6.880, *p* < 0.001); and (c) among the subtypes of inhibitory neurons, the highest morphological similarity was observed for connections linking neurons within the 5HT3aR, followed by the PV and Sst (*p* < 0.05).

## DISCUSSION

This study proposed a method to construct microscopic brain networks based on neuronal morphological similarity. We demonstrated several nontrivial global topological configurations that were shared across species. Locally, hub nodes were disproportionately located in specific classes and showed unique neuronal morphology, and connections between classes contributed considerably more to bridge edges. Neuronal morphological similarity of intraclass connections was higher than interclass connections, which further differed among classes (deeper > superficial layers, inhibitory > excitatory neurons, and isocortical > subcortical structures). Intriguingly, neuronal morphological similarity was related to neuronal axonal projections. Finally, a more segregated but less integrated, resilient, and economic network architecture was observed for the human than mouse. Overall, these findings advance our understanding of brain network organization at the microscopic level.

### Optimized Topology of Microscopic Morphological Brain Networks

We found optimized topological organization in all microscopic morphological brain networks, including small-worldness, economy, resilience, and heavy-tailed degree distribution. These findings suggest that the general principles of cortical development and the basic architecture of the cortex are conserved across mammals, and are consistent with previous brain network studies in terms of chemical and electrical synapses (Gal et al., 2017; Schröter et al., 2017; Shih et al., 2015; Varshney et al., 2011) and based on neuroimaging techniques (Bullmore & Sporns, 2012; Liao et al., 2017; Lynn & Bassett, 2019; Scharwächter et al., 2022; Zhang et al., 2022). Interestingly, we showed that the small-worldness and economy held for neurons in each cortical layer, cell type, and brain region, indicating a fractal, self-similar organization of microscopic morphological brain networks. Self-similarity has been demonstrated for different aspects of the brain, ranging from neuronal dendritic structures (Takeda et al., 1992) and spiking patterns (Guo et al., 2020) to large-scale brain networks (Zheng et al., 2020). Accordingly, our findings together with previous results collectively suggest that the brain has evolved into an optimized wiring layout across spatial scales, presumably as a consequence of natural selection to satisfy the balance between minimizing biological cost and maximizing information transmission efficiency (Bassett & Bullmore, 2006; Bullmore & Sporns, 2009).

### Effects of Cortical Layers, Cell Types, and Brain Regions on Microscopic Morphological Brain Networks

We found profound effects of cortical layers, cell types, and brain regions on microscopic morphological brain networks. First, significantly higher morphological similarity was observed for intra- than interclass connections. This is not surprising given that neurons in different classes have different soma locations, morphologies, and projection patterns (Adesnik & Naka, 2018; Callaway, 1998; DeFelipe et al., 2013; de Kock et al., 2007; Kawaguchi & Kubota, 1997). Furthermore, we found that the levels of intraclass morphological similarity differed among classes with the deep layers, inhibitory neurons, and isocortical structures displaying higher values than their corresponding counterparts. The higher morphological similarity of inhibitory than excitatory neurons is consistent with previous findings that inhibitory neurons exhibit higher firing rates and form stronger synaptic connections than excitatory neurons (Barth & Poulet, 2012). Finally, we showed that different subtypes of inhibitory neurons with distinct molecular markers exhibited different levels of morphological similarity. Altogether, our findings indicate that interneuron morphological similarity is dependent on the cytoarchitectonic, chemoarchitectonic, and laminar organization of neurons and differs between regions with different evolutionary timelines.

We also demonstrated the dependence of hub nodes and bridge edges on cortical layers, cell types, and brain regions. Specifically, neurons in superficial layers, inhibitory neurons, and neurons in subcortical structures made disproportionate contributions to hub nodes. Previous studies showed that superficial layers in the rat somatosensory cortex received diverse inputs, distributed massive cortico-cortical connections, and were crucial for context-dependent sensory processing and sensorimotor integration (Diamond et al., 2008; Petreanu et al., 2012; Xu et al., 2012). For inhibitory neurons, it is well known that they are crucial for cortical information processing by gating signal flows and influencing network dynamics (Tremblay et al., 2016). In the developing hippocampal networks, hub neurons were mainly composed of inhibitory neurons in both the rat and mouse (Bonifazi et al., 2009). With respect to subcortical structures, their participation in a highly connected set of regions were consistently reported in the rat, mouse, and human (Cole et al., 2010; Liska et al., 2015; van den Heuvel et al., 2016). Based on preferential attachment models of network growth, which suggest that newly added nodes in a network tend to connect with existing nodes with a high number of links (Bell et al., 2017; Poncela et al., 2008), it is reasonable to observe hub nodes in subcortical structures since these regions are the older, more primitive part of the brain from an evolutionary perspective. Overall, our findings provide novel evidence from the viewpoint of neuronal morphology-based coordination that superficial layers, inhibitory neurons, and subcortical structures play central roles in coordinating information flow in brain networks.

For bridge edges, interclass connections made a disproportionate contribution in microscopic morphological brain networks. This is consistent with neuroimaging-based findings that bridge edges are mainly composed of intermodule connections (He et al., 2009). Given that hub regions primarily comprise connectors with many intermodule connections, we speculate that the central roles of superficial layers, inhibitory neurons, and subcortical structures may be played by their interactions with other classes.

### Unique Neuronal Morphology of Hub Nodes in Microscopic Morphological Brain Networks

We found that hub nodes exhibited unique neuronal morphologies, indicating the prominent role of neuronal morphology in determining how neurons function and communicate (Elston, 2000; Koch & Segev, 2000; Poirazi & Mel, 2001). Specifically, hub neurons in both the rat somatosensory and mouse whole-brain networks displayed smaller values for many features related to neuronal spatial size, such as soma surface and neuronal width and height. The more compact spatial embedding of the hub neurons may be an evolutionary adaptation for energy-efficient signal transmission. Moreover, the common pattern of unique neuronal morphology of hub neurons between rat and mouse may be attributed to shared genetic expression and similar cytoarchitecture in evolutionarily close species (Bjerke et al., 2021; Espinosa et al., 2009; Prasad et al., 2013). For the human MTG network, however, only a few features differentiated hub from nonhub neurons, possibly due to the limited number of neurons used for network construction. Interestingly, hub nodes in all microscopic morphological brain networks exhibited higher values for the angle between the compartment of a bifurcating father branch and its two daughter branches. These findings suggest that hub nodes have more divergent branching regardless of species, which presumably enables them to connect with a broad range of neurons in the brain. This sounds plausible given findings from neuroimaging studies that hub regions have diverse connectivity profiles and properties (van den Heuvel & Sporns, 2013).

### Relationship Between Neuronal Morphological Similarity and Axonal Projection

In this study, we observed a significantly positive correlation in the pattern of interregion similarity of connectivity profiles between morphological similarity and axonal projection. This is consistent with previous macroscopic neuroimaging studies in animals and humans, which demonstrated that morphological similarity was significantly related to axonal connectivity (Li et al., 2024; Sebenius et al., 2023; Seidlitz et al., 2018; Wang et al., 2024). These macroscopic findings, together with our microscopic observations, collectively suggest that morphological similarity may serve as a potential indicator of structural connectivity. This notion aligns with a previous structural model relating cortico-cortical connections to laminar differences between linked areas (Barbas, 2015; García-Cabezas et al., 2019). Based on this model, Zhang et al. (2020) found that anatomical centrality, a proxy for the laminar differentiation gradient, contributed to the topological organization of structural brain networks centrality. This is in line with our findings that laminar differentiation significantly affected interneuron morphological similarity and the distribution of hubs. Nevertheless, inconsistencies were also observed between neuronal morphological similarity and axonal projection networks. For example, only one region was commonly identified as a hub for the two types of networks. In addition, the more compact spatial embedding of hub neurons observed in this study was contrasted with previous findings from structural brain networks that hubs had long connectivity lengths (Li et al., 2023; van den Heuvel et al., 2012). Thus, morphological similarity is not equivalent to structural connectivity and offers a unique framework for a broader understanding of the brain network organization.

### Different Microscopic Morphological Brain Networks Between Human and Mouse

We found that the human MTG neurons exhibited a more segregated but less integrated, economic, and resilient network architecture than the mouse VISp neurons. These findings suggest that although the mouse VISp are often considered as a comparator for the human MTG, the extent to which their neurons are optimally organized differs largely from those in the human MTG. The differences may be due to differential cellular compositions between the human MTG and mouse VISp neurons, such as the size and density of neurons and the diversity of molecularly defined glutamatergic t-types (Berg et al., 2021; Hodge et al., 2019). The higher segregation and lower integration suggest a shift toward a more regular-like network configuration for the human MTG than the mouse VISp neurons, which minimizes the wiring cost at the expense of losing global integrative capacity and is vulnerable to targeted attacks (Bonilha et al., 2014; Bullmore & Sporns, 2012). This shift may be related to the engagement of specific cognitive abilities (Barbey, 2018). Compared with other mammalian brains, the human brain tremendously expands its cognitive repertoire, such as unique language abilities. Previous studies have shown that the MTG is involved in language-related processing (Heyer et al., 2022; Hickok & Poeppel, 2007; Ojemann et al., 2002). We thus speculate that the distinct network architecture in the human MTG may result from evolution, favoring specialized language processing in humans. Notably, our findings should be explained with caution as only an extremely limited number of neurons were used to construct the networks. Additionally, it is important to recognize that the observed cross-species differences may be biased due to different regions used for the comparisons. Future studies should examine the cross-species differences using more homologous regions between species.

### Limitations and Future Directions

First, we extracted morphological features from reconstructed neurons to build microscopic brain networks. However, the reconstruction may suffer from errors due to factors such as image quality, algorithm accuracy, and manual operation, which may lead to inaccuracies in the estimation of the interneuron similarity. Second, the neuron reconstruction data were extremely limited and from a small patch of a single brain region. Thus, whether our findings can be generalized is not known given structural and functional heterogeneity within and across brain regions. Third, although it is important to examine cross-dataset reproducibility of our results, this is challenging from a practical perspective, as no similar datasets exist that match those used in this study in terms of species, neuronal distribution, and experimental conditions. Future studies are needed to examine the reproducibility of our results using independent datasets. Finally, brain networks are essentially directed since neurons can excite or inhibit other neurons. However, our proposed method cannot capture this directed connectivity information.

## Acknowledgments

This work was supported by the grant from Research Center for Brain Cognition and Human Development, Guangdong, China (No. 2024B0303390003), National Natural Science Foundation of China (No. 82472092), STI 2030—Major Projects (No. 2021ZD0200500), National Social Science Foundation of China (No. 20&ZD296), Key-Area Research and Development Program of Guangdong Province (2019B030335001).

## Supporting Information

Supporting information for this article is available at https://doi.org/10.1162/netn_a_00458.

## Author Contributions

Suhui Jin: Data curation; Formal analysis; Methodology; Visualization; Writing – original draft. Junle Li: Data curation; Writing – review & editing. Jinhui Wang: Conceptualization; Investigation; Resources; Supervision; Writing – review & editing.

## Funding Information

Jinhui Wang, Research Center for Brain Cognition and Human Development, Guangdong, China, Award ID: 2024B0303390003. Jinhui Wang, National Natural Science Foundation of China (https://dx.doi.org/10.13039/501100001809), Award ID: 82472092. Jinhui Wang, STI 2030—Major Projects, Award ID: 2021ZD0200500. Jinhui Wang, National Social Science Fund of China (https://dx.doi.org/10.13039/501100012456), Award ID: 20&ZD296. Jinhui Wang, Key-Area Research and Development Program of Guangdong Province, Award ID: 2019B030335001.

## Data Availability Statement

All data that support the findings of this study are from publicly available datasets.

## Supplementary Material



## TECHNICAL TERMS

Clustering coefficient: The extent of local cliquishness of nodes in a network, reflecting local density of edges.
Characteristic path length: The average shortest path length between all pairs of nodes in a network, reflecting overall communication efficiency.
Degree distribution: The probability distribution of the number of connections each node has in a network.
Small-worldness: A combination of high clustering coefficient and short characteristic path length, enabling both localized and global information processing.
Cost efficiency: A measure of how effectively a network minimizes wiring cost while maintaining efficient information transfer.
Hubs: Highly connected nodes that play central roles in maintaining network communication and integration.
Resilience: The ability of a network to maintain functionality when nodes or edges are removed.
