# Supplementary material for: Brain connectome from neuronal morphology
Source: Netw Neurosci. 2025 Jul 29;9(3):913–37. doi: 10.1162/netn_a_00458 (PMC12543300; doi:10.1162/netn_a_00458)
Supplement: Supplementary file 1 [file netn-9-3-913-s001.pdf]

Supplementary Materials for

Brain connectome from neuronal morphology

Supplementary Figures

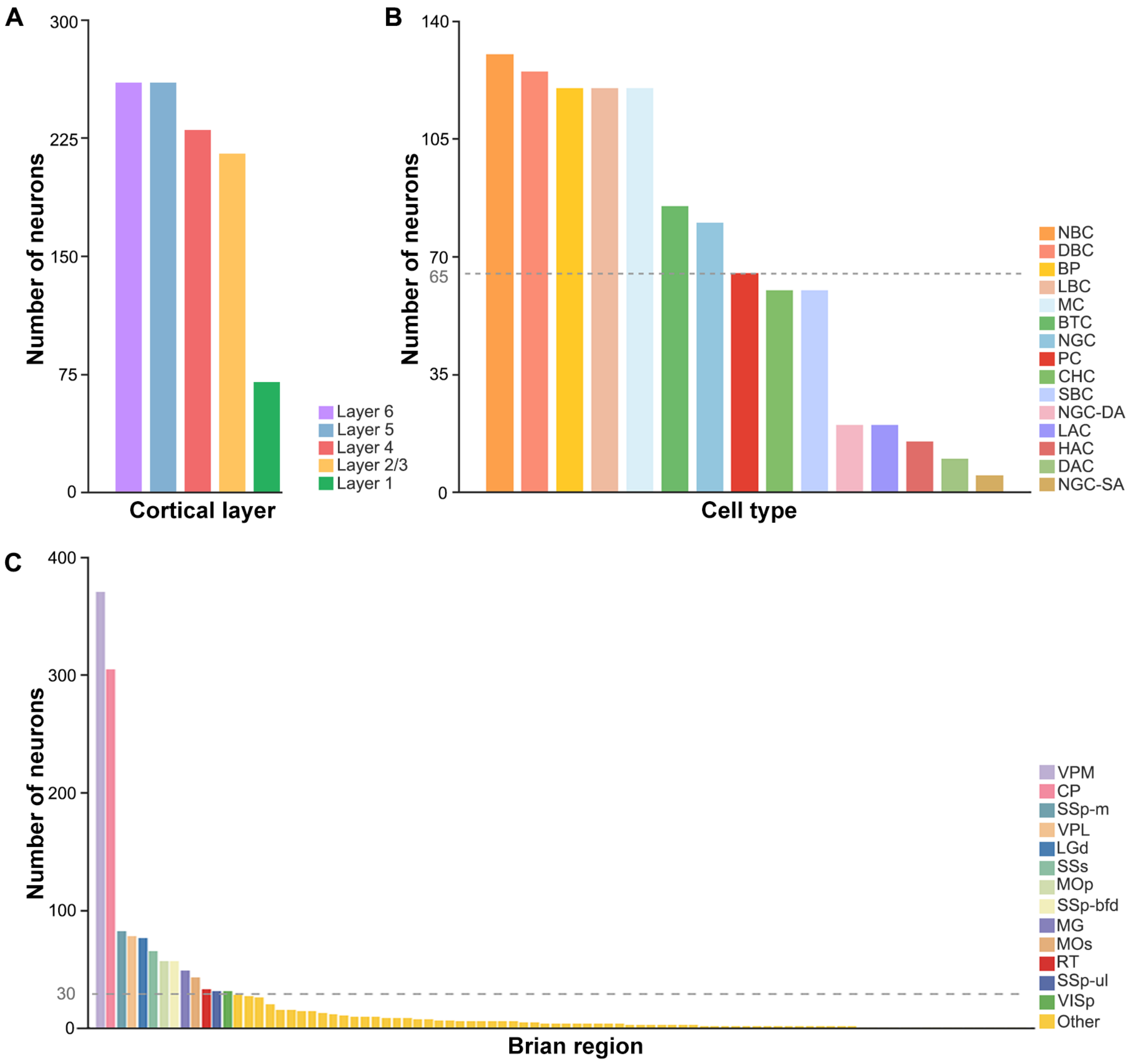

Figure S1. The number of neurons in different cortical layers, cell types, and brain regions. (A) The number of neurons in different cortical layers in the dataset 1. (B) The number of neurons in different cell types in the dataset 1. (C) The number of neurons in different brain region in the dataset 2. The grey dashed lines represent the cut-off values above which the cell types or brain regions were included to examine their effects on the rat somatosensory and mouse whole-brain microscopic morphological brain networks. NBC, nest basket cell;

DBC, double Bouquet cell; BP, bipolar cell; LBC, large basket cell; MC, Martinotti cell; NGC, neurogliaform cell; BTC, bitufted cell; PC, pyramidal cell; CHC, chandelier cell; SBC, small basket cell; NGC-DA, neurogliaform cell with dense axonal arborization; LAC, large axon cell; DAC, descending axon cell; NGC-SA, neurogliafomm cell with slender axonal arborization; VPM, ventral posteromedial nucleus; CP, caudoputamen; SSp-m, primary somatosensory area, mouth; VPL, ventral posterolateral nucleus; LGd, lateral geniculate complex, dorsal partd; SSs, supplemental somatosensory area; Mop, primary motor area; SSp-bfd, primary somatosensory barrel cortex; MG, medial geniculate complex; MOs, secondary motor area; RT, reticular nucleus; SSp-ul, primary somatosensory area, upper limb; VISp, primary visual area.

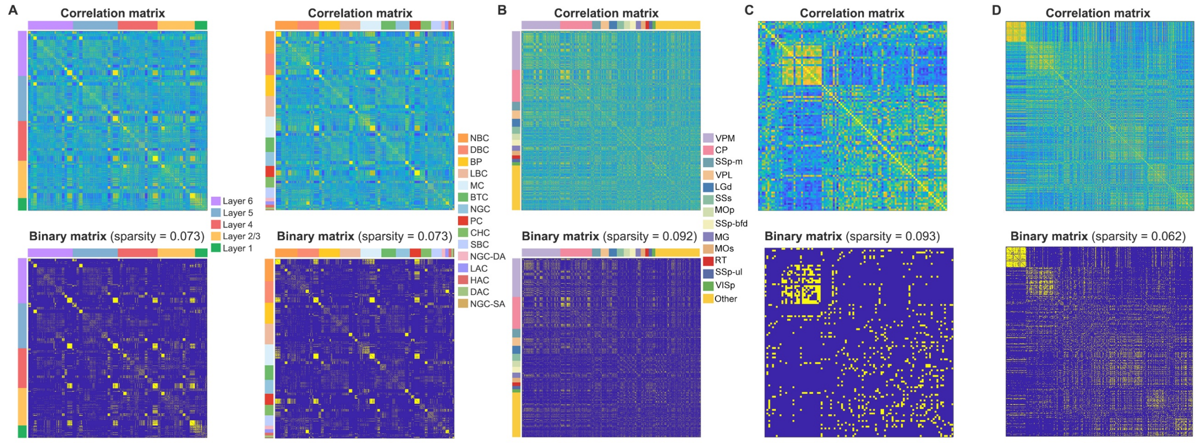

Figure S2. Inter-neuron correlation matrices and corresponding binary networks of the rat somatosensory neurons (A), mouse whole-brain neurons (B), human MTG neurons (C), and mouse VISp and TEa neurons (D). The correlation matrices were obtained by calculating the Pearson correlation across 42 morphological features between neurons, and were binarized to sparse networks with no isolated nodes. The correlation matrix and binary network of the rat somatosensory neurons were arranged in terms of the cortical layers (A, left) and cell types (A, right) of the neurons, respectively. NBC, nest basket cell; DBC, double Bouquet cell; BP, bipolar cell; LBC, large basket cell; MC, Martinotti cell; NGC, neurogliaform cell; BTC,

bitufted cell; PC, pyramidal cell; CHC, chandelier cell; SBC, small basket cell; NGC-DA, neurogliaform cell with dense axonal arborization; LAC, large axon cell; DAC, descending axon cell; NGC-SA, neurogliafomm cell with slender axonal arborization; VPM, ventral posteromedial nucleus; CP, caudoputamen; SSp-m, primary somatosensory area, mouth; VPL, ventral posterolateral nucleus; LGd, lateral geniculate complex, dorsal partd; SSs, supplemental somatosensory area; Mop, primary motor area; SSp-bfd, primary somatosensory barrel cortex; MG, medial geniculate complex; MOs, secondary motor area; RT, reticular nucleus; SSp-ul, primary somatosensory area, upper limb; VISp, primary visual area.

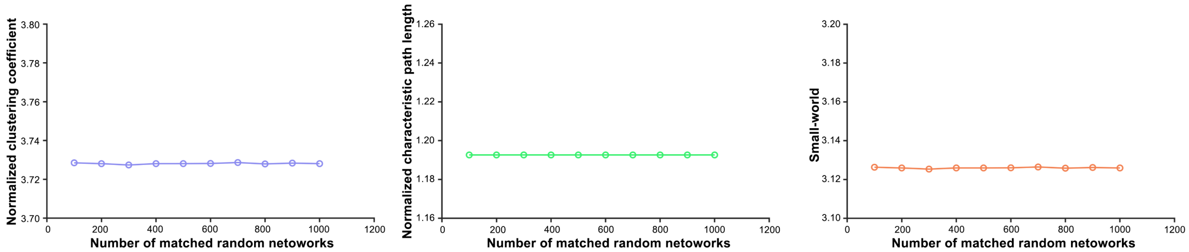

Figure S3. Robustness of the small-world organization against varying numbers of random networks. When different numbers of random networks were used, small fluctuations in the estimates of normalized clustering coefficient and normalized characteristic path length were observed for the mouse whole-brain microscopic morphological brain network, which contained the most nodes in this study.

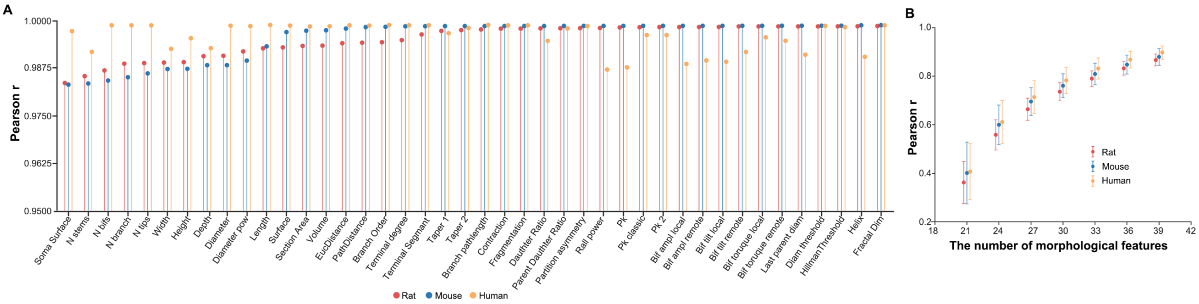

Figure S4. Stability and robustness of microscopic morphological brain networks. (A) Using a leave-one-feature-out approach, high Pearson correlation coefficients were observed between the inter-neuron correlation matrices derived from all morphological features and those generated with one feature removed. (B) As the number of morphological features increased, the inter-neuron correlation matrices became increasingly similar to the inter-neuron correlation matrices derived from all morphological features. Particularly, when more than 30 morphological features were used, the resulting inter-neuron correlation matrices exhibited high Pearson correlation coefficients (approximate or greater than 0.8) with those derived from all morphological features.

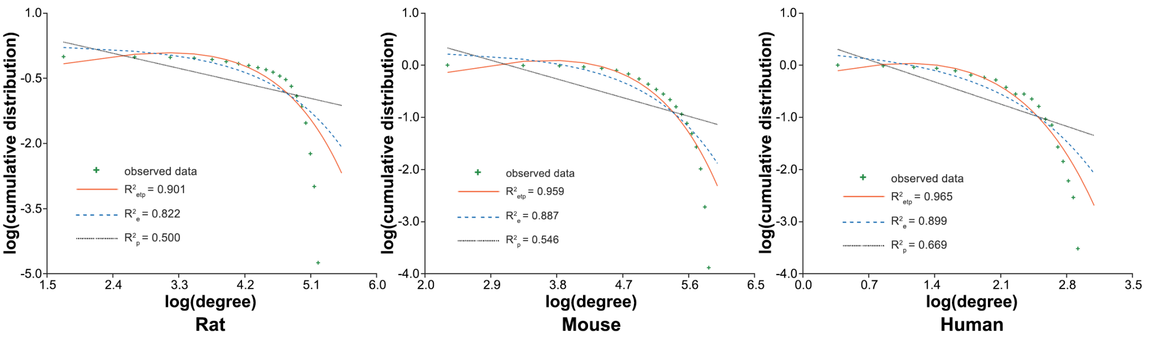

Figure S5. Degree distributions of the rat somatosensory, mouse whole-brain, and human MTG microscopic morphological brain networks. The degree distributions of all microscopic morphological brain networks were best fitted by exponentially truncated power laws. This type of degree distribution suggests the existence of highly connected hub neurons in the microscopic morphological brain networks. The orange, blue dotted and black dotted lines indicate the fits of the exponentially truncated power law [$\mathrm{P(k)} \sim k^{\alpha-1} e^{-\frac{k}{k_c}}$], exponential power law [$\mathrm{P(k)} \sim e^{-x/x_c}$], and power law [$\mathrm{P(k)} \sim x^{\alpha-1}$], respectively. R^2_{etp}, goodness-of-fit for an exponentially truncated power law; R^2_e, goodness-of-fit for an exponential distribution; R^2_p, goodness-of-fit for a power law.

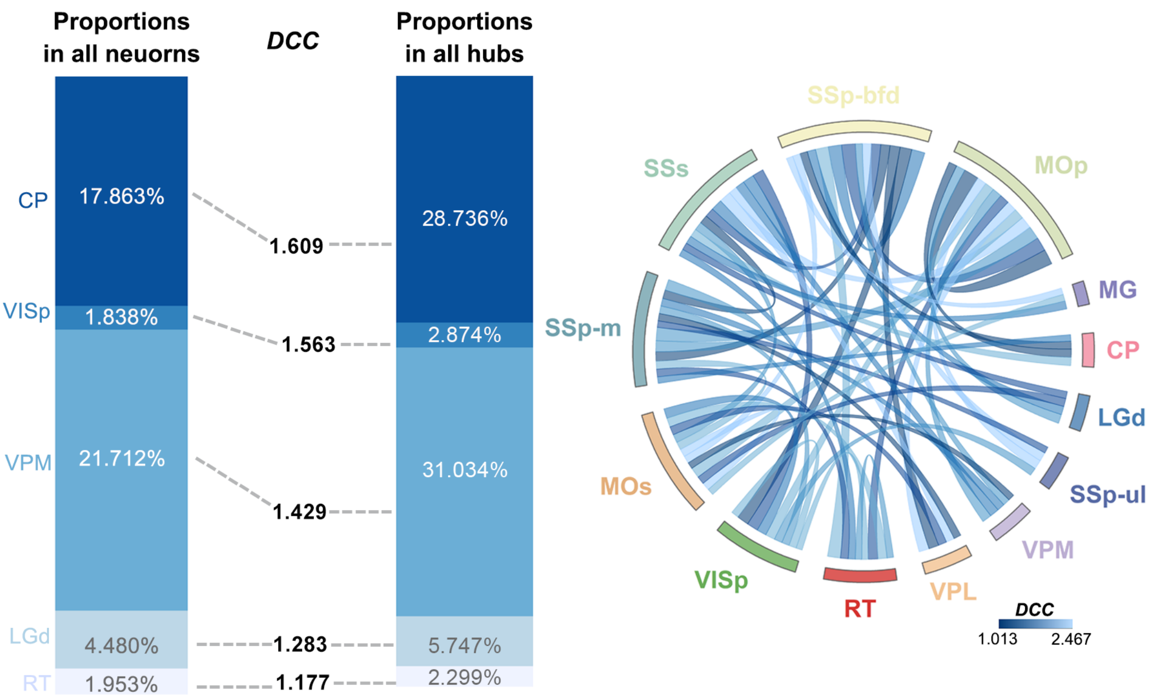

Figure S6. Distribution of hub nodes and bridge edges in the mouse whole-brain microscopic morphological brain network. (A) The hub nodes were disproportionately located in the CP, VISp, VPM, LGd, and RT. (B) The bridge edges preferred connections linking different classes. Figure S6B shows the connections between 46 pairs of brain regions and within 7 brain regions made disproportionate contributions to the bridge edges (i.e., *DCC* > 1). *DCC*, disproportionate contribution coefficient. VPM, ventral posteromedial nucleus; CP, caudoputamen; SSp-m, primary somatosensory area, mouth; VPL, ventral posterolateral nucleus; LGd, lateral geniculate complex, dorsal partd; SSs, supplemental somatosensory area; Mop, primary motor area; SSp-bfd, primary somatosensory barrel cortex; MG, medial geniculate complex; MOs, secondary motor area; RT, reticular nucleus; SSp-ul, primary somatosensory area, upper limb; VISp, primary visual area.

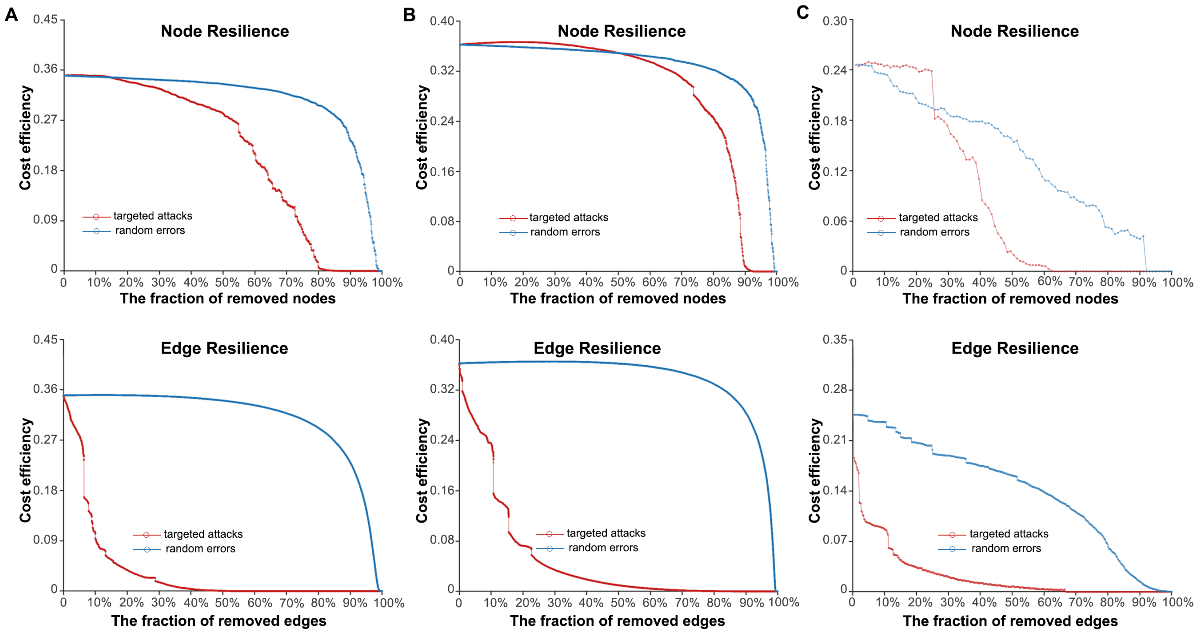

Figure S7. Resilience to removal of nodes and edges in the rat somatosensory (A), mouse whole-brain (B), and human MTG (C) microscopic morphological brain network. Compared with random errors, targeted attacks on nodes with high degree and on edges with high betweenness led to more rapid decline in the cost efficiency of the rat somatosensory microscopic morphological brain network.

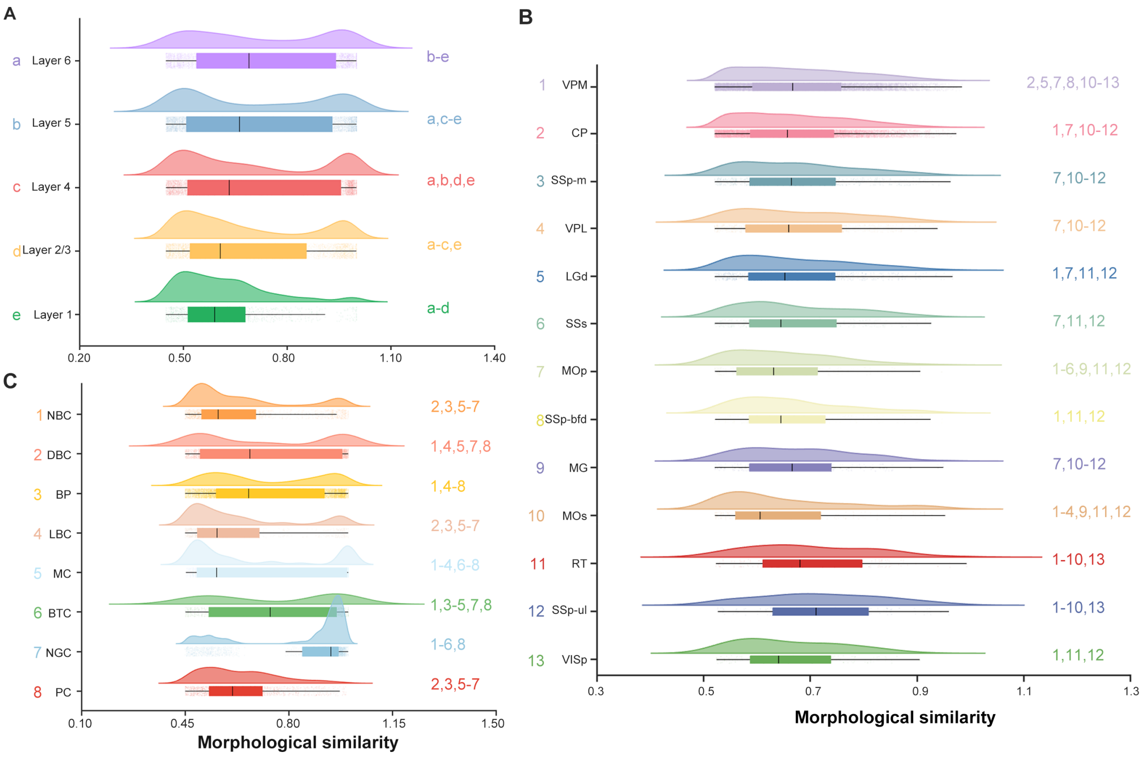

Figure S8. Differences in morphological similarity between different cortical layers, cell types, and brain regions. (A) For cortical layers, a continuous increase in the inter-neuron morphological similarity was observed from the superficial to the deep layers. (B) With regard to cell types, different levels of inter-neuron morphological similarity were observed, with the highest value for NGC neurons and the lowest value for MC neurons. (C) Different brain regions exhibited different levels of inter-neuron morphological similarity, with the highest value within the SSp-ul and lowest value within the MOs. The letters/numbers on the left side of the left/right panel indicate different cortical layers/cell types/brain regions, and the letters/numbers on the right side of the left/right panel indicate the cortical layers/cell types/brain regions from which the morphological similarity of a given cortical layer/cell type/brain regions differed. NBC, nest basket cell; DBC, double Bouquet cell; BP, bipolar cell; LBC, large basket cell; MC, Martinotti cell; NGC, neurogliaform cell; BTC, bitufted cell; PC, pyramidal cell; VPM, ventral posteromedial nucleus; CP, caudoputamen; SSp-m, primary somatosensory area, mouth; VPL, ventral posterolateral nucleus; LGd, lateral

geniculate complex, dorsal partd; SSs, supplemental somatosensory area; Mop, primary motor area; SSp-bfd, primary somatosensory barrel cortex; MG, medial geniculate complex; MOs, secondary motor area; RT, reticular nucleus; SSp-ul, primary somatosensory area, upper limb; VISp, primary visual area.

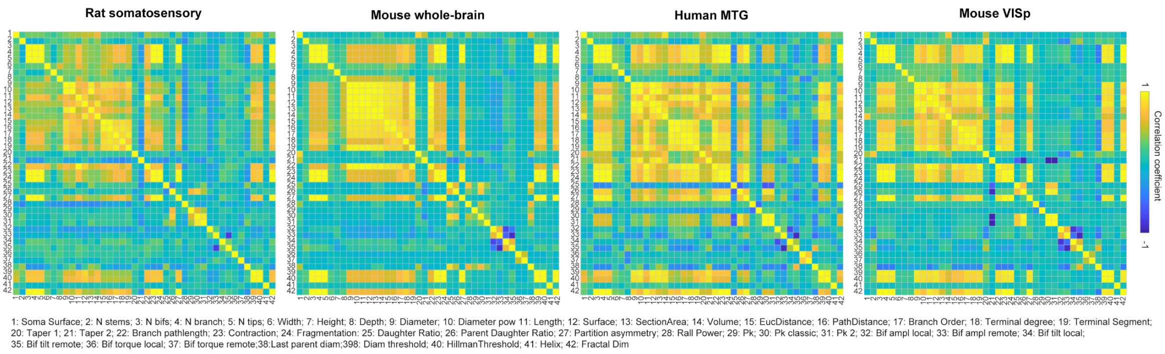

Figure S9. Relationship between neuronal morphological features. Strongly correlations were observed between certain pairs of neuronal morphological features regardless of the species, such as fragmentation and partition asymmetry.

Supplementary Table

Table S1. Morphological features used for construction of microscopic morphological brain networks

Features	Description	Statistics
Soma_Surface	Surface of the soma	Total sum
N_stems	Number of stems attached to the soma	Total sum
N_bifs	Total number of bifurcations	Total sum
N_branch	Total number of branches	Total sum
N_tips	Number of terminal tips for the given input neuron	Total sum
Width	Width of neuron	Total sum
Height	High of neuron	Total sum
Depth	Depth of neuron	Total sum
Diameter	Diameter of each compartment the neuron	Total sum
Diameter_pow	Diameter raised to the power 1.5 for each compartment	Total sum
Length	Total arborization length	Total sum
Surface	Surface of the compartment	Total sum
SectionArea	Section area of the compartment	Total sum
Volume	Volume of the compartment	Total sum
EucDistance	Maximum Euclidean distance from soma to the tips	Total sum
PathDistance	Path distance of a compartment	Total sum
Branch_Order	Order of the branch with respect to soma	Total sum
Terminal_degree	Total number from each compartment to termination tip	Total sum
Terminal Segment	Terminal branch	Total sum
Taper_1	Burke taper	Total sum
Taper_2	Hillman taper	Total sum
Branch_pathlength	Sum of the length of all compartments	Total sum
Contraction	Ratio between Euclidean distance of a branch and its path length	Total sum
Fragmentation	Total number of compartments that constitute a branch between two bifurcation points	Compartments
Daughter_Ratio	Ratio between the bigger daughter and the other one	Average
Parent_Daughter_Ratio	Ratio between the diameter of a daughter and its father	Average
Partition_asymmetry	Average over all bifurcations of sub-trees	Total sum
Rall_Power	The best value that fits Rallís power	Average
Pk	Ratio of (d1^rall+d2^rall)/(bifurcDiam^rall)	Average
Pk_classic	The same value as Pk, but with Rall_Power sets to 1.5	Average

Pk_2	The same value as Pk, but with Rall_Power sets to 2	Average
Bif_ampl_local	Angle between the first two bifurcation compartments	Average
Bif_ampl_remote	Angle between current plane of bifurcation and previous plane of bifurcation	Average
Bif_tilt_local	The smaller of the angles between the previous compartment of bifurcating father and the two daughter branches of the same bifurcation	Average
Bif_tilt_remote	Angle between the previous father compartment of the current bifurcating father and its two daughter compartment	Average
Bif_torque_local	Angle between the plane of previous bifurcation and the current bifurcatio	Average
Bif_torque_remote	Angle between current plane of bifurcation and previous plane of bifurcation	Average
Last_parent_diam	Diameter of last bifurcation before the terminal tips	Average
Diam_threshold	Diameter of first compartment after the last bifurcation leading to a terminal tip	Total sum
Hillman Threshold	Weighted average between 50% of father and 25% of daughter diameters of the terminal bifurcation	Total sum
Helix	Helix by choosing the 3 segments at a time (or four points at a time) and computes the normal form on the 3 vectors to find the 4th vector	Total sum
Fractal_Dim	Slope of linear fit of regression line obtained from the plot of path vs. Euclidean distances	Total sum
